# Mechanical Analysis and Corrosion Analysis of Zinc Alloys for Bioabsorbable Implants for Osteosynthesis

**DOI:** 10.3390/ma15020421

**Published:** 2022-01-06

**Authors:** Salome Hagelstein, Sergej Zankovic, Adalbert Kovacs, Roland Barkhoff, Michael Seidenstuecker

**Affiliations:** 1 G.E.R.N. Tissue Replacement, Regeneration & Neogenesis, Department of Orthopedics and Trauma Surgery, Medical Center-Albert-Ludwigs-University of Freiburg, Faculty of Medicine, Albert-Ludwigs-University of Freiburg, Hugstetter Straße 55, 79106 Freiburg, Germany; sergej.zankovic@uniklinik-freiburg.de (S.Z.); michael.seidenstuecker@uniklinik-freiburg.de (M.S.); 2 Limedion GmbH, Coatings and Surface Analysis, Am Schäferstock 2-4, 68163 Mannheim, Germany; kovacs@limedion.de; 3 Quadralux e.K., Am Schäferstock 2-4, 68163 Mannheim, Germany; r.barkhoff@quadralux.de

**Keywords:** biodegradation, zinc alloy, mechanical stability, tensile testing, zinc silver, zinc titanium, corrosion testing

## Abstract

Zinc alloys have recently been researched intensely for their great properties as bioabsorbable implants for osteosynthesis. Pure zinc (Zn) itself has relatively poor strength, which makes it insufficient for most clinical use. Research has already proven that the mechanical strength of zinc can be enhanced significantly by alloying it with silver. This study evaluated zinc silver alloys (ZnAg) as well as novel zinc silver titanium alloys (ZnAgTi) regarding their mechanical properties for the use as bioabsorbable implants. Compared to pure zinc the mechanical strength was enhanced significantly for all tested zinc alloys. The elastic properties were only enhanced significantly for the zinc silver alloys ZnAg6 and ZnAg9. Regarding target values for orthopedic implants proposed in literature, the best mechanical properties were measured for the ZnAg3Ti1 alloy with an ultimate tensile strength of 262 MPa and an elongation at fracture of 16%. Besides the mechanical properties, the corrosion rates are important for bioabsorbable implants. This study tested the corrosion rates of zinc alloys in PBS solution (phosphate buffered solution) with electrochemical corrosion measurement. Zinc and its alloys showed favorable corrosion rates, especially in comparison to magnesium, which has a much lower degradation rate and no buildup of hydrogen gas pockets during the process. Altogether, this makes zinc alloys highly favorable for use as material for bioabsorbable implants for osteosynthesis.

## 1. Introduction

Especially in the last two decades, research for bioabsorbable implants has been intensified [1,2,3,4] because they offer great advantages in comparison to conventional implants. Conventional implant materials are associated with many adverse effects like stress shielding [5,6,7], chronic inflammation [8,9], soft tissue damage, and stress on the body through metallosis [8,10,11]. Bioabsorbable implants include materials that not only degrade within the body but also offer no harm to the body during corrosion and focus especially on how they affect the host’s metabolism [12]. In the search for bioabsorbable implants for osteosynthesis, there is still a lot of room for improvement. There is an ongoing search for implants, which exhibit mechanical properties that match those of bone and offer the sufficient strength needed. Mechanical stability is critical for fracture healing, as a great stability in connection with only a minimal volume change of the fracture gap and without shearing of the fragments leads to optimal conditions for bone healing [13,14]. Reviews have discussed different target values for bone implants. Targets for ultimate tensile strength have been set between 200 and 300 MPa, an elongation at fracture of around 15% to 20% and a Young’s modulus of 10 to 30 GPa [15,16,17,18,19]. Venezuela et al. [20], for instance, proposed target values of a yield strength of >230 MPa, a tensile strength of >300 MPa, a Young’s modulus of 10–20 GPa, and an elongation at fracture of 15–18% for orthopedic internal fixation devices.

During the research for bioabsorbable implants interest has been increasingly drawn to zinc alloys as zinc exhibits favorable mechanical properties regarding the use as bioabsorbable implants, while also showing reasonable low corrosion rates [21,22,23]. Furthermore, zinc is not converted to hydrogen during its degradation process, as is the case with magnesium, where there have been issues with hydrogen accumulating in the surrounding tissue and, therefore, possible adverse effects on the process of bone healing [11,24,25]. With an ultimate tensile strength of 100 to 150 MPa [26,27] and a small Young’s modulus, zinc exhibits properties that are close to those of bone [27]. Notably, the strength of zinc is considerably lower than the required target values of implants for osteosynthesis. There has been a significant effort put into researching the process of alloying zinc to gain higher strength values and more favorable mechanical properties for the use as orthopedic implants [28,29]. For instance, Sikora-Jasinska et al. [30] showed that the ultimate tensile strength of zinc could be increased over 200 MPa when alloyed with silver, while also keeping its beneficial elastic properties.

Regarding its use as a bioabsorbable implant, zinc is also expected to show good biocompatibility [28,31]. It is closely regulated by zinc transporters and metallothioneins and is one of the most abundant essential elements in the body [32,33]. A daily intake of about 8 to 11 mg is recommended for adults, whereas the maximum daily intake of zinc is limited to 40 mg/day [34,35]. Additionally, studies have shown positive effects of zinc on cell proliferation, differentiation, and mineralization of extracellular matrix as well as bone growth [36,37,38,39,40].

For a better suitability of zinc as a material for implants for osteosynthesis it can be alloyed with different metals. In this study we focused on zinc alloys with silver and titanium. Titanium is the most used metal for implants in orthopedics today. It has been used since the 1950s and it has been studied intensively [41]. Its biocompatibility with a slow corrosion rate and bioinertness have been already established in literature [42]. Silver has also been used as an implant material in humans for a long time and has shown a very good biocompatibility [43]. Silver is also an essential trace element and it is recommended to consume 0.4 to 27 μg of silver daily [44]. As early as 1924, Zierold et al. [45] proved that silver is excellently suited for implantation in bone. In this study, an improved regeneration of the bone occurred in the cancellous bone, while the cortical regeneration was not influenced. Furthermore, it was demonstrated that silver nanoparticles improved fracture healing in the rat model [46]. In addition, silver has a direct antibacterial effect and is used in therapy for particularly resistant bacteria [43,47,48]. In this study, novel zinc alloys were biomechanically analyzed and evaluated for future use as bioabsorbable implants in orthopedic fixation devices. Therefore, mechanical properties were measured and compared to target values as well as mechanical properties of implants already available for clinical use like MgYERZr (Syntellix AG, Hannover, Germany). Additionally, corrosion measurements were performed in order to get an idea of the corrosion characteristics of the novel zinc alloys.

## 2. Materials and Methods

### 2.1. Tensile Testing

This study used tensile tests to determine the tensile strength of zinc alloys in comparison to pure zinc. Dog bone-shaped test specimens were manufactured by Limedion GmbH., Mannheim, Germany, according to ISO standard 6892-1:2019 [49], with a grain size of <15 µm. Testing was carried out at room temperature with the material testing machine Z005 (ZwickRoell, GmbH & Co. KG, Ulm, Germany) under position control with a nominal stress of 30 MPa $s^{-1}$ until failure. Failure was defined as an increase of strain by 500% to the previous measuring point or a stress drop to 2% of the maximum stress. Strain–stress curves were recorded with TestXpert3 software, Version 3.31 (ZwickRoell, GmbH & Co. KG, Ulm, Germany) and used to analyze ultimate tensile strength (UTS) and yield strength (YS). Elongation at fracture (A) was calculated by measuring the test specimens after breakage with the stereomicroscope Olympus SZ61 (Olympus K.K., Shinjuku, Japan) and the software ImageJ (FIJI modification). Tensile testing was carried out with Zn, ZnTi0.5, ZnAg6, ZnAg9, ZnAg1Ti1, and ZnAg3Ti1 alloys.

### 2.2. Corrosion Measurements

#### 2.2.1. Samples

**Figure 1:**
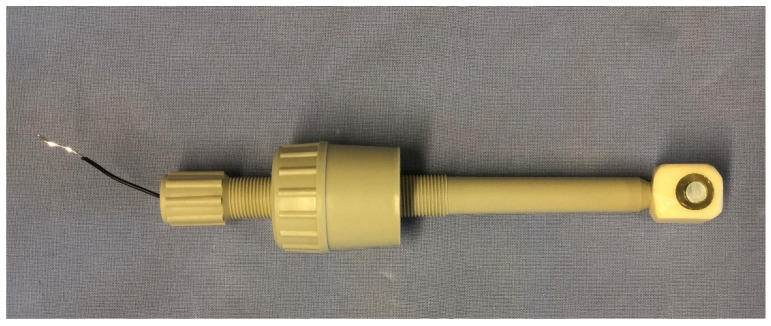
Corrosion sample attached to sample holder.

The electrochemical measurement was carried out on cylinders of the different alloys (Zn, ZnTi0.5, Zn3, ZnAg6, ZnAg1Ti1 and ZnAg3Ti1) with the dimensions d = 6 mm and h = 10 mm, with a surface area of 0.2826 cm². The samples were each fixed as watertight in a specially made sample holder made of FC52 polyol and FC52 isocyanate (see Figure 1). The samples were placed in this mold with a precise fit and attached to the measuring apparatus using the mold. Each sample was measured twice with 10 min between measurements, and the mean was calculated.

#### 2.2.2. Electrochemical Testing

**Figure 2:**
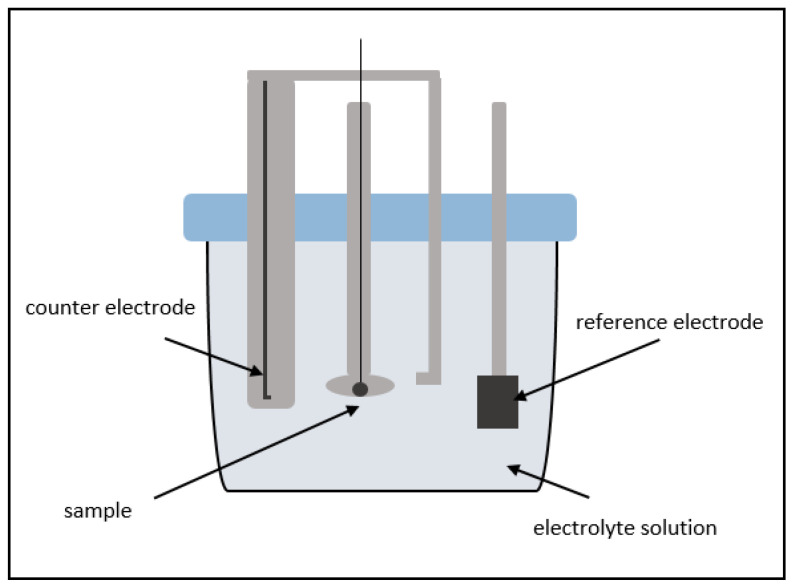
Schematic layout of the corrosion measuring cell.

To determine the corrosion rate of the prepared samples, potentiodynamic polarization tests were carried out in PBS solution (8 g/L NaCl, 0.2 g/L KCl, 1.44 g/L Na_2_HPO_4_ and 0.245 g/L KH_2_PO_4)_) at 37 ± 2 °C and pH = 7.4 ± 0.2. The electrochemical measurement was carried out with a three-electrode measuring system, with a potentiostat/galvanostat PS2000, the corrosion measuring cell KMZ5 from Sensortechnik Meinsberg (Xylem Analytics Germany Sales GmbH & Co. KG, Waldheim, Germany), and the Ag/AgCl reference electrode SE11. A 2 cm × 2 cm Pt foil (Xylem Analytics Germany Sales GmbH & Co. KG, Waldheim, Germany) was used as counter electrode (see Figure 2). Immediately before the measurements all samples were polished with 1200 SiC (silicon carbide) paper.

**Figure 3:**
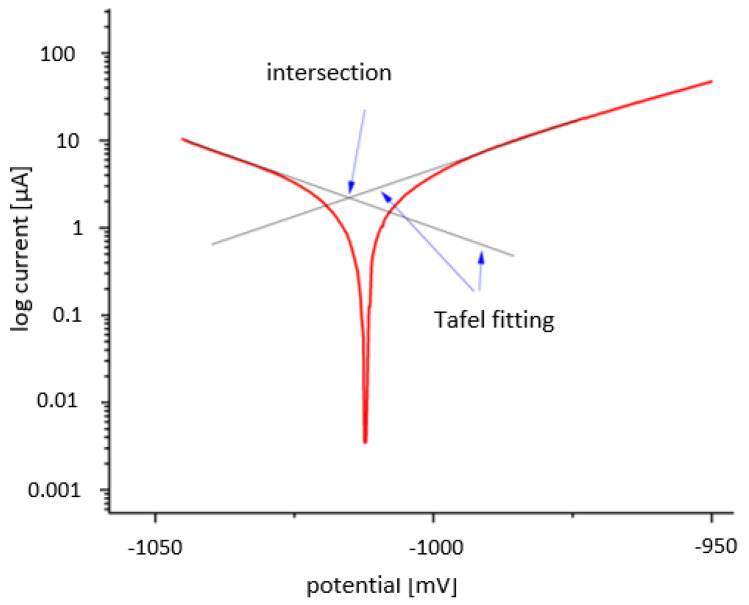
Example of a Tafel fitting on log current-potential curve.

Measurements were conducted at a 0.25 mV scan rate with ±50 mV around the rest potential. Open circuit potential (OCP) was measured for 30 min before the corrosion measurement to ensure the potential stability of the studied system. The corrosion current and the corrosion potential were defined as the intersection of two straight lines. For this purpose, Tafel fittings were applied to the linear sections of the log current-potential curve of the individual measurements in Origin Pro 2020 (OriginLab, Northampton, MA, United States) and their intersection point was determined (see Figure 3).

Using Faraday’s law, the corrosion rate was calculated with the following equation:

$$R_{M}=\frac{M}{\mathrm{nF}\rho }i_{\mathrm{corr}}$$

where M is Atomic mass, n is the number of electrons involved in the reaction, ρ is density, and F is 96,485 C/mol.

This was calculated for each alloy accordingly (see Table 1).

### 2.3. Statistical Analysis

Statistical analysis was conducted with Microsoft Office Excel^®^, Version 2013 (Microsoft Cooperation, Redmond, WA, United States) and Origin Pro 2020 (OriginLab, Northampton, MA, United States). All quantitative data were given as mean ± standard deviation. Statistical significance was determined with unpaired one-way analysis of variance (ANOVA) and the significance level was set to *p* < 0.05.

## 3. Results

### 3.1. Tensile Testing

The mechanical properties of zinc and its alloys are shown in Table 2. Comparison revealed an increase of ultimate tensile strength (UTS) as well as elongation at fracture [A]. Elongation at fracture was only enhanced significantly in comparison to pure zinc for the zinc silver alloys ZnAg6 and ZnAg9 with *p* = 0.009 and *p* = 3.09 × $10^{-4}$ respectively. The UTS was increased significantly (*p* < 0.05) for all alloys.

**Figure 4:**
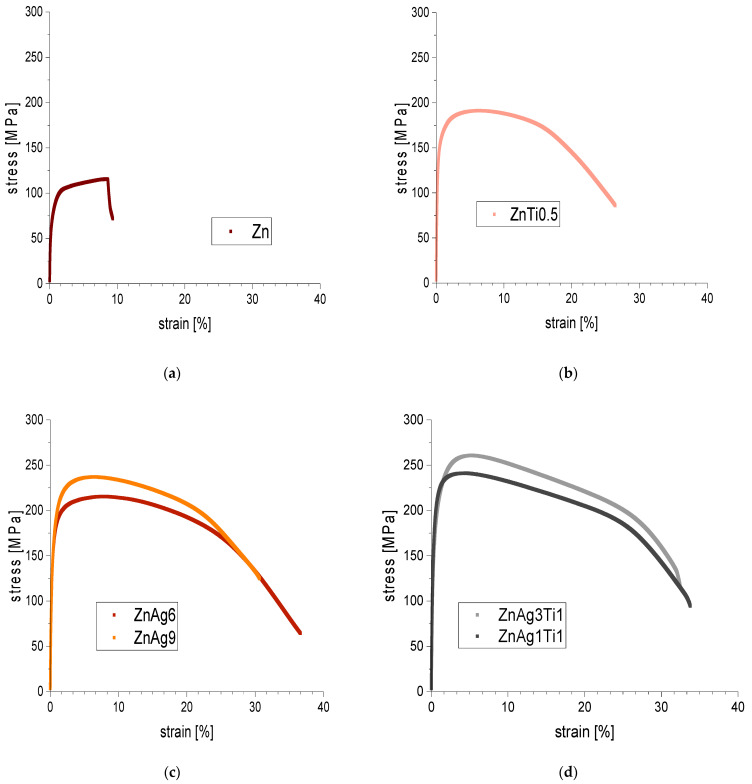
Averaged stress–strain curves of all tensile tests with the ZwickRoell Z005 materials testing machine and recorded with TestXpert3, with (**a**) pure zinc, (**b**) ZnTi0.5 alloy, (**c**) zinc silver alloys, and (**d**) zinc silver titanium alloys.

As seen by comparing the stress–strain curves shown in Figure 4, the ultimate tensile strength was also enhanced with increasing silver content. The alloys ZnAg9 and ZnAg3Ti1 showed higher tensile strengths compared to ZnAg6 and ZnAg1Ti1, respectively. This increase was significant for ZnAg9 in comparison to ZnAg6 (*p* = 0.02) but not for ZnAg3Ti1 in comparison to ZnAg1Ti1 (*p* = 0.06). Ultimate tensile strength was also enhanced significantly for ZnAg3Ti1 in comparison to the zinc silver alloys (ZnAg6 *p* = 1.5 × $10^{-5}$ and ZnAg9 *p* = 0.048).

### 3.2. Corrosion Measurement

**Figure 5:**
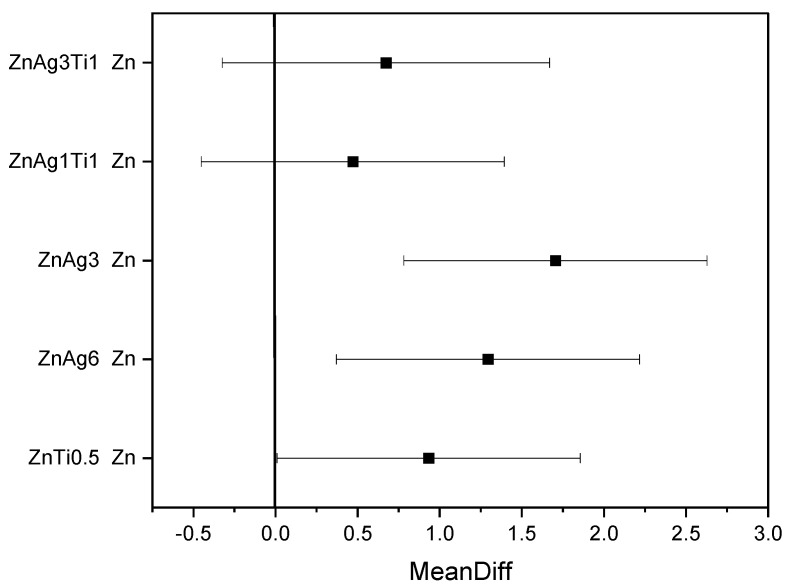
Mean difference of corrosion current after ANOVA between zinc and the tested alloys.

The corrosion measurements are used as an indication of the corrosion processes happening in the body. Table 3 shows the results of the electrochemical corrosion testing. Corrosion rates of zinc were accelerated by alloying it with titanium or silver. This was significant for ZnAg3, ZnAg6, and ZnTi0.5 in comparison to pure zinc with *p* < 0.05 (see Figure 5). The corrosion rates of the ZnAgTi alloys were not significantly different to the corrosion rate of pure zinc (see Figure 5). The highest corrosion rates were measured for the ZnAg alloys with a corrosion rate of 0.11 mm/year for ZnAg6 and 0.14 mm/year for ZnAg3, respectively.

## 4. Discussion

### 4.1. Tensile Testing

This study verified that alloying zinc with silver increases the tensile strength in comparison to pure zinc significantly. Tensile strength rose with increasing content of silver for both zinc silver alloys as well as zinc titanium silver alloys. This is in accordance with previous studies.

In this study, we measured an ultimate tensile strength of pure zinc of 115 MPa with an elongation at fracture of 10%. Xiao et al. [50] reported very similar properties with an ultimate tensile strengths of 110 MPa and an elongation at fracture of 15% for pure zinc in dog bone-shaped specimens.

For zinc silver alloys, Sikora-Jasinska et al. [30] described, for example, a tensile strength of 203 MPa for ZnAg2.5 alloys and a tensile strength of 287 MPa for ZnAg7 alloys. These values correspond to the results of our study, with a tensile strength of ZnAg6 of about 211 MPa and for ZnAg9 of about 233 MPa. A similar tensile strength was also reported by Yang et al. [51], with a tensile strength of ZnAg0.8 of 190 MPa and for ZnAg2 of 240 MPa. Both studies reported an increase of strength with higher silver content of the alloys. The difference in the values can be explained by different manufacturing techniques. The test specimens we used were manufactured by Limedion (Limedion GmbH., Mannheim, Germany), which were hot extruded at 325 °C with an extrusion rate of 25:1 and showed a grain size of <15 µm. Sikora-Jasinska et al. [30] used specimens that were hot extruded at 250 °C with an extrusion rate of 14:1, whereas Yang et al. [51] tested specimens, which were hot extruded at 260 °C with an extrusion rate of 36:1 and showed a grain size of <10 µm. Hot extrusion is known to enhance the mechanical properties of metal alloys as it refines the grain size and homogenizes the alloy [29]. Homogenous alloys with smaller grain size showed more favorable mechanical properties [8]. This could be one explanation for the higher mechanical properties measured by Yang et al. [51] in comparison to our measurements.

The elastic properties of zinc in our tests were only significantly altered by alloying with silver for ZnAg6 and ZnAg9. There was no significant enhanced elongation at fracture for the zinc silver titanium alloys. The measured elastic values for the tested zinc silver alloys can also be confirmed by comparing it to literature. Sikora-Jasinska et al. [30] and Yang et al. [51] reported elongation at fracture of 30% to 40% for their tested zinc silver alloys. The zinc silver alloys tested by us showed an elongation at fracture of 25 to 30% for ZnAg6 and ZnAg9, which are quite similar.

The zinc titanium alloys had a slightly lower elongation at fracture with 16 to 21% in our measurements. These values are very close to and, for ZnAg3Ti1, even meet the target values proposed by Venezuela et al. [20] for orthopedic fixation devices. Additionally, the tested zinc silver titanium alloys showed even greater mechanical strength properties compared to the zinc silver alloys. ZnAg1Ti1 and ZnAg3Ti1 tested for an ultimate tensile strength of 241 and 262 MPa, respectively.

In comparison to the mechanical target values suggested in the literature, the ZnAg3Ti1 and ZnAg6 alloys showed excellent properties for the values we measured. They both met an ultimate tensile strength of 200 to 300 MPa, while also possessing good elastic properties with a reasonably low elongation at fracture. ZnAg3Ti1 could also meet the criteria of an elongation at fracture of 15 to 18% with a measured elongation of 16%.

**Figure 6:**
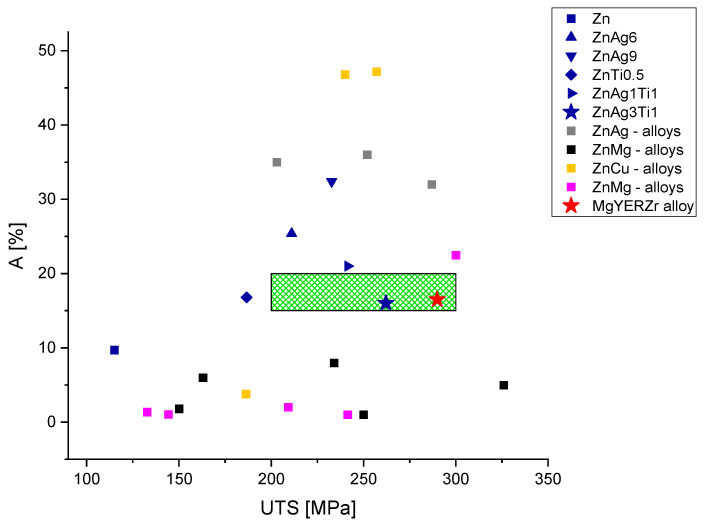
Comparison of elongation at fracture (A) and ultimate tensile strength (UTS) of the alloys measured in this study (blue icons) with measurements reported in the literature [30,52,53,54,55,56,57,58]. The area highlighted in green corresponds to the target values given in the literature of an elongation at fracture between 15 to 20% and an ultimate tensile strength between 200 to 300 MPa.

Our zinc silver titanium alloys achieved a combination of good mechanical stability as well as favorable elastic properties. This makes them also stand out in comparison to tested zinc alloys in the literature, as seen in Figure 6. In comparison to the reported mechanical values of bioabsorbable alloys and also in regards of the MgYERZr alloys already in clinical use, the ZnAg3Ti1 alloy displayed especially excellent mechanical properties.

**Figure 7:**
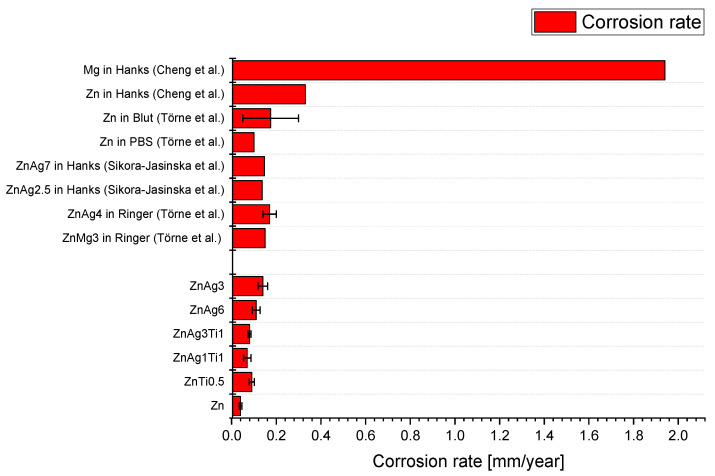
Corrosion rates of zinc and zinc alloys measured in electrochemical corrosion testing in different media [21,22,30,31].

Both ZnAg1Ti1 and ZnAg3Ti1 showed a good ultimate tensile strength as well as a reasonably low elongation at fracture. The only two alloys achieving the target values of 15 to 20% elongation at fracture, as well as an ultimate tensile strength between 200 and 300 MPa, were the ZnAg3Ti1 alloy tested in this study and the MgYERZr alloy used for the MAGNEZIX Pin from Syntellix (Syntellix AG, Hannover, Germany) [57,58] (see Figure 7). This makes the zinc silver titanium alloys suitable for further testing for bioabsorbable implants. Their biocompatibility as well as their corrosion rates are important for future clinical use and need to be evaluated in future testing.

### 4.2. Corrosion Measurements

The measurements showed that the corrosion rates of the zinc alloys can be significantly influenced by the composition of the alloys (see Figure 5). Furthermore, all measured corrosion rates proved to be significantly lower than the corrosion rates of the magnesium alloys [52,59]. Thus, the zinc alloys had a better corrosion resistance than magnesium alloys.

For example, Vojtech et al. [52] reported a significantly higher corrosion tendency for magnesium in contrast to zinc. They described a resting potential for zinc of 0.89 V in SBF with a pH of 7. For magnesium, they reported a resting potential of –1.64 V. The resting potential of zinc was higher than that of magnesium. The resting potential of zinc in our measurements was 0.97 V and, thus, was in a similar order of magnitude.

Törne et al. [21] reported corrosion rates in PBS solution of zinc of less than 0.1 mm/year. This corresponds closely to the corrosion rate of pure zinc of about 0.04 mm/year measured during our experiment (see Figure 7). Additionally, Hehrlein et al. [60] measured the immersion corrosion rate of ZnAg3 alloy in SBF (simulated body fluid) over a period of 60 days, where they measured a corrosion rate of 0.16 mm/year. This is also very close to the corrosion rate measured by us in PBS.

The corrosion rates of pure zinc can be increased by alloying zinc with silver. For example, the corrosion rate increased for the zinc silver alloys measured by us. We measured the highest corrosion rates for ZnAg3 with 0.14 mm/year. For ZnAg6 the corrosion rate decreased slightly to 0.11 mm/year instead.

Sikora-Jasinska et al. [30] also measured the corrosion rates for zinc and zinc silver alloys in electrochemical corrosion tests in Hank’s solution. For zinc, they determined a resting potential of −0.98 V and a corrosion rate of 0.133 mm/year. As the silver content increased, so did the corrosion rates. Thus, the corrosion rate for ZnAg2.5 was about 0.137 mm/year and for ZnAg7, 0.147 mm/year. Static immersion with subsequent determination of the weight also showed an increasing corrosion rate with increasing silver content. The increased corrosion rate of the zinc-silver alloys resulted, among other things, from the formation of two phases by alloying silver with zinc. This resulted in localized increased corrosion rates on the ZnAg3 phases with increased cathodic reaction rates [22].

This different dynamic of the corrosion rates with increasing silver content in comparison to our measurement can possibly be explained by the use of a different corrosion medium. The use of PBS led to less corrosion on average and the alloys had the opportunity to repassivate through the phosphate-based buffer [8]. For example, the corrosion rates in Törne et al. [22] were 0.1 mm/year for pure Zn and 1.7 mm/year for ZnAg4 in Ringer’s solution (see Figure 7). This is higher than the corrosion rate of the zinc silver alloys we measured. Zaludin et al. [61] also showed that the corrosion rates changed greatly when SBF (simulated body fluid), Ringer’s solution, or PBS were used. For example, the corrosion currents of magnesium increased from 11.19 μA in PBS to 236.05 μA in SBF and 676.87 μA in Ringer solution. Li et al. [59] described corrosion rates of zinc in SBF of about 0.102 mm/year and in Gamble’s solution (GS) of 0.034 mm/year. Correlating to this, the surfaces of the zinc samples in SBF showed more significant corrosion damage than the zinc samples in GS.

Another reason for the different dynamics of the corrosion rates could be that the ZnAg3 alloys had a larger grain size due to the manufacturing process and, thus, an increased corrosion rate. The corrosion rates in the measurement can be influenced by the processing, microstructure, and especially the grain size of the alloy. A particularly smooth surface, a homogeneous alloy, and the smallest possible grain size led to lower corrosion rates [2]. A change in grain size from 400 μm to 10 μm can reduce the corrosion rate by up to 50% [19]. This also needs to be considered in evaluating the corrosion rates.

The dynamics of the corrosion rates for zinc silver alloys with increasing silver content is, thus, not yet conclusively solved.

The electrochemical corrosion tests represent an important building block in the research for new materials for bioabsorbable implants. General requirements for bioabsorbable stents were summarized in a table by Bowen et al. [17]. Here they claimed that corrosion rates of 0.02 mm/year are required. In the review by Venezuela et al. [20] a corrosion rate of approx. 0.5 mm/year is required for bioabsorbable plates and screws. Generally, implanted screws must maintain mechanical integrity for 6 months to a certain degree and then dissolve completely within 1 to 2 years. The zinc alloys we investigated had corrosion rates of 0.04 mm/year to 0.14 mm/year, which is within the range of corrosion rates required in the literature for bioabsorbable implants. The electrochemically measured corrosion rates can also be approximated in comparison with in vivo tests.

Bowen et al. [28], for example, investigated the corrosion rates of zinc by implanting zinc wires into the abdominal aorta of rats. Uniform corrosion rates for zinc were demonstrated for the first 3 months. Localized corrosion damage, such as pitting, did not form on the zinc implants until 4.5 and 6 months. They also reported a corrosion rate of 0.02 to 0.05 mm/year. These in vivo corrosion rates correlate with the corrosion rates we measured for zinc of 0.04 mm/year in the electrochemical measurements; thus, it can be assumed that the measured corrosion rates of the zinc alloys may also be transferred to the in vivo experiments and corrode homogeneously at the beginning.

Kafri et al. [23] also measured a corrosion rate within the first 14 weeks of pure zinc implanted subcutaneously in rats of approximately 0.06 mm/year in in vivo experiments.

Xiao et al. [50] confirmed the homogeneous corrosion and biocompatibility of zinc alloys in bone. With a corrosion rate of 0.15 mm/year, the implants showed good osteointegration already after 12 weeks. Therefore, it can be assumed that the zinc alloys we tested may also achieve the desired corrosion properties while implanted in bone. This still must be confirmed in corresponding tests.

For magnesium alloys, Huehnerschulte et al. [62] already examined magnesium alloys with rare earth elements, similar to the magnesium alloy of the Syntellix products, in in vivo tests in 2011. The calculated corrosion rates of the ZEK100 and AX30 alloys were 0.07 mm/year for the first 3 months. Thus, the corrosion rates of the zinc alloys measured by us with, for example, 0.08 mm/year for the ZnAg3Ti1 alloy or 0.11 mm/year for the ZnAg6 alloy are also in similar orders of magnitude compared to magnesium alloys with rare earths. With that, they achieved similar corrosion properties like bioabsorbable alloys that are already in clinical use.

For a correct assessment, additional investigations should be carried out in vivo, as the corrosion properties differ significantly depending on the implantation site and the environmental milieu [50,63]. Furthermore, especially for clinical utility, an assessment of the change in mechanical properties under corrosion is needed to better evaluate the mechanical integrity of the implant. For example, Huehnerschulte et al. [62] showed that the various magnesium alloys exhibited significant differences in flexural strength after corrosion, despite the same corrosion rates. For zinc, studies here showed that aging and creep processes could lead to an impairment of the mechanical properties [29]. Kafri et al. [23] also showed that the corrosion rate weakened significantly over time. This requires further investigation after corrosion in vitro and in vivo.

## 5. Conclusions

Zinc and its alloys are promising candidates for further investigation as materials for bioabsorbable implants for osteosynthesis. ZnAg6 and ZnAg3Ti1 showed excellent mechanical values in tensile testing. With a UTS of above 200 MPa, they met the criteria mentioned in literature for implants for osteosynthesis. These mechanical properties and their corrosion rates, which are significantly lower as common magnesium alloys, make them highly favorable for clinical use. We could prove that the corrosion rates of these alloys also lay within target values mentioned in literature for bioabsorbable implant materials. With the analysis of these alloys, novel zinc alloys are brought into the focus for further investigation for bioabsorbable implants. These findings make them promising candidates, but they still must be investigated further, especially with clinical trials.

## Figures and Tables

**Table 1:** Values for calculating the corrosion rate per year for each element according to Faraday’s law.

Element	M [g/mol]	n	ρ [g/cm³]	Rmicorr[mm/year]
Zn	65.38	2	7.14	0.05
Ti	47.87	4	4.51	0.03
Ag	107.87	2	10.49	0.06

**Table 2:** Mechanical properties of zinc and zinc alloys during tensile testing according to DIN ISO 6892-1:2019.

Materials		Mechanical Properties	
	Elongation [%]	UTS [MPa]	YS [MPa]
Zn	9.72 ± 7.87	114.98 ± 4.87	110.66 ± 6.79
ZnTi0.5	16.81 ± 1.57	186.71 ± 3.40	189.06 ± 2.04
ZnAg6	25.39 ± 4.08	211.06 ± 11.58	213.78 ± 6.59
ZnAg9	32.40 ± 6.44	232.75 ± 10.55	232.64 ± 10.19
ZnAg1Ti1	20.99 ± 2.98	241.46 ± 1.00	240.41 ± 0.53
ZnAg3Ti1	16.00 ± 3.55	262.11 ± 4.98	261.59 ± 5.14

**Table 3:** Measurements of the corrosion testing with mean and standard deviation.

Materials	OCP [mV]	Ecorr. [mV]	Icorr. [µA]	Corrosion Rate [mm/year]
Zn	−967 ± 2.50	−959 ± 6.24	0.85 ± 0.11	0.04
ZnTi0.5	−998 ± 16.62	−1002 ± 14.24	1.78 ± 0.22	0.09
ZnAg3	−993.25 ± 12.63	−1000.5 ± 15.95	2.55 ± 0.40	0.14
ZnAg6	−977 ± 19.08	−965 ± 20.86	2.14 ± 0.34	0.11
ZnAg1Ti1	−996.75 ± 14.93	−999 ± 12.73	1.32 ± 0.31	0.07
ZnAg3Ti1	−982.25 ± 8.30	−979.67 ± 18.23	1.52 ± 0.12	0.08

## Data Availability

The data presented in this article are available on request from the corresponding author.
